# Supplementary material for: Unexpected High Species Diversity of Mesolycus Gorham (Coleoptera, Lycidae) from China, with a Preliminary Investigation on Its Phylogenetic Position Based on Multiple Genes
Source: Insects. 2022 Dec 17;13(12):1171. doi: 10.3390/insects13121171 (PMC9782631; doi:10.3390/insects13121171)
Supplement: Supplementary file 1 [file insects-13-01171-s001.zip › insects-2071643-supplementary.pdf]

Supplementary File

Unexpected high species diversity of *Mesolycus* Gorham (Coleoptera, Lycidae) from China, with a preliminary investigation on its phylogenetic position based on multiple genes †

Haoyu Liu [1,*], Ruolan Du [1], Ya Kang [1], Xueying Ge [1], Xingke Yang [2] and Yuxia Yang [1,*]

[1] Key Laboratory of Zoological Systematics and Application, School of Life Science, Institute of Life Science and Green Development, Hebei University, Baoding 071002, China
[2] Key Laboratory of Zoological Systematics and Evolution, Institute of Zoology, Chinese Academy of Sciences, Beijing 100101, China
* Correspondence: liuhy@hbu.edu.cn (H.L.); yxyang@hbu.edu.cn (Y.Y.)
† This published work and the nomenclatural acts it contains have been registered in ZooBank, the Online Registration System for the ICZN (International Code of Zoological Nomenclature). The LSID (Life Science Identifier) for This Publication is: LSID urn:lsid:zoobank.org:pub:ADBEC6C2-5FD6-4182-9AC4-D1717348E1AE.

Table S1. The distribution information of *Mesolycus* species

Species	Longitude	Latitude	Location	References
M. mediozonatus (Nakane, 1955)	139.469	35.606	Japan, Mt. Zohzu, Kagawa	[9]
	138.747	35.561	Japan, Mt. Hohoh, Yamanashi	[9]
M. holzschuhi (Bic, 2002)	90.140	23.300	NE India, Menghalaya, 3 km E of Tura	[10]
	89.740	27.490	W-Bhutan, Thimphu Distr., E of Dochu-la, Menshunang	[10]
M. shaanxiensis sp. n.	103.544	31.108	China, Shaanxi, Ningshan, Huoditang	This study
	107.843	33.852	China, Shaanxi, Zhouzhi, Houzhenzi	This study
M. hubeicus (Kazantsev, 2004)	110.300	31.500	China, W Hubei, Dashennongjia	[9]
M. varus sp. n.	99.222	27.331	China, Yunnan, Weixi, Pantiange	This study
M. sausai (Bic, 2002)	101.210	21.070	Laos, 15 km NW Louang Namtha	[10]
M. bhutanensis (Bic, 2002)	89.750	27.491	W-Bhutan, Thimphu Distr., E of Dochu-la, Menshunang	[10]

M. vitalisi (Pic, 1923)	103.775	22.304	Vietnam, Mts W Chapa, Fan-Si-Pan	[9]
M. breviplatus sp. n.	98.700	27.936	China, Yunnan, Dimaluo - Biluoxueshan	This study
M. murzini Kazantsev, 2004	94.596	23.597	N Myanmar (Burma), 50 km E Putao, env. Nan Thi	[9]
	96.031	28.717	China, Xizang, Zayu, Shang Zayu	This study
M. atrorufus (Kiesenwetter, 1879)	139.690	36.219	Japan, Nankuril Island	[41]
	138.041	36.228	Japan, Hokkaido	[41]
	133.670	33.803	Japan, Honshu	[41]
	130.791	32.686	Japan, Shikoku	[41]
	138.253	36.205	Japan, Kyushu	[41]
	133.637	33.743	Japan, Tsukojima	[41]
	129.722	31.721	Japan, Yakushima	[41]
	130.514	30.355	Japan, Tanegashima	[41]
	147.600	45.197	Japan, Chugoku region	[41]
M. nanensis (Kazantsev, 2004）	101.070	19.130	N Thailand, Nan Prov., Dai Phu Kha	[9]
M. dentatus sp. n.	100.568	22.060	China, Yunnan, Menglongbanna, Mengsong	This study
M. berezowskii (Kazantsev, 2000)	103.826	36.059	China, Gansu	[8]
M. laosensis (Bic, 2002)	103.040	18.230	Laos, Saisombun zone, Phou Khao Khouay N. P., Tad Leuk	[10]
M. particularis (Pic, 1928) comb. n.	85.324	27.717	Nepal, Kathmandu, Godavari	[10]
	105.467	21.369	N Vietnam, Tam Dao	[10]
M. atricollis (Pic,1926) comb. n.	120.695	23.506	Taiwan, Fenchihu	[10]
	120.437	24.152	Taiwan, Tapang (1800), Taichung-hsien	[10]
	120.666	23.299	Taiwan, Alishan	[10]
M. qinlinganus (Kazantsev, 2000)	106.390	35.412	China, Ningxia, Jingyuan, Erlonghe Forestry	This study
	105.116	32.735	China, Gansu,Fanba	This study
	104.150	33.150	China, Gansu, Qiujiaba	This study
	104.417	34.435	China,Zhouqu, Shatan Forestry	This study
	107.245	33.300	China, China, Shaanxi, Quinling Shan, 35 km S of Baoji	[10]

	100.110	27.200	China, Yunnan prov., Jinsha riv., Daju	[10]
	110.300	31.500	China, W Hubei prov., Dashennongjia Nat. Res., Muyu	[10]
	107.325	34.064	China, Shaanxi prov., QingLing Shan mts., road Baoji, Taibai vill.	[10]
	100.160	27.130	China, N Yunnan, Yulongshan mts., E slope,	[10]
	98.540	28.060	China, Yunnan, Hengduan mts.	[10]
	101.050	28.070	China, NW Sichuan, 30 km NW Muli, Bowa, mixed forest, 3500 m	[10]
	104.932	33.398	China, Gansu, Minshan	[8]
	100.140	27.070	China, Gansu, W Wudu	[8]
M. tibetanus (Kazantsev, 2000)	103.322	30.606	China, Sichuan, Xiling snow Mts.	[8]
	99.026	28.440	China, NW Yunnan, E Weixi, Yunling Mt. Range.	[8]
M. pygmaeus (Waterhouse, 1879)	114.554	0.962	Borneo	[9]
M. ater (Pic, 1943)	102.077	2.210	Borneo	[9]
M. shelfordi (Bourgeois, 1906)	116.073	5.998	Borneo, Kina-Balu-Geb	[9]
	109.329	-0.402	Borneo, Pontianak, Borneo	[9]
M. obscurus (Pic, 1912)	116.591	5.666	N Borneo, Kinabalu	[9]
	102.260	0.500	E Sumatra, Riau Prov., Bukit Tigapuluh N.P.	[9]
M. ilyai (Kazantsev, 2000)	101.877	29.596	China, Sichuan, Xiling snow Mts.	[8]
	103.229	30.708	China, Moxi, east of Gongashan	[8]
M. pacholatkoi (Bic, 2002) comb. n.	98.986	18.798	N Thailand, Changmai	[10]
	105.111	26.817	China, Guizhou, Dakua, 35 km NE Leishan	[10]
	101.247	20.941	Laos, Louangnamtha prov.	[10]
	98.760	25.408	N Vietnam, Tam Dao	[10]
	98.164	19.520	China, Yunnan, Gaoligong mts.	[10]
M. fedorenkoi (Kazantsev, 2013)	108.400	12.100	S Vietnam, Lam Dong Prov., Bi DoupNui Ba Nat. Reserve, env. Long Lanh	[13]
M.rubromarginatus (Kazantsev, 2013)	104.010	20.120	E Laos, Hua Phan Prov., Ban Saleui, Phou Pan Mt.	[13]
	100.568	22.060	China, Yunnan, Menglongbanna, Mengsong	This study

M. discoidalis (Pic, 1912)	116.590	5.665	Borneo	[9]
M. jendeki (Bic, 2002)	106.350	15.020	Laos, Attapu prov., Bolaven Plateau, 15 km SE of Ban Houaykong, Nong Lom lake env.	[10]
M. bolavensis (Bic, 2002)	106.350	15.020	Laos, Attapu prov., Bolaven Plateau, 15 km SE of Ban Houaykong, Nong Lom lake env.	[10]

Table S2. The primers of *COI* gene used for PCR

Sequence Method	Gene	Primer	Sequence	Reference
Sanger sequencing	*COI*	LCO1490	5′-GGTCAACAAATCATA AAGATATTGG-3′	[20]
Sanger sequencing		HCO2198	5′-TAAACTTCAGGGTGACCAAAAAATCA-3′	[20]

Table S3. Information on the mitochondrial gene fragments of the Lycidae species used in this study

	Tribe	Species	Locality information	Lrna	COI	COII	ND5	Reference
Ingroup	Ateliini	*Scarelus anthracinus*	Malaysia	HM451002	HM451042	HM451042*	HM451211	[4]
		Scarelus pseudoumbosus	Malaysia	HM450999	HM451038	HM451038*	HM451207	[4]
	Calochromini	*Micronychus* sp.	RSA	KU495971	KU496109	KU496109*	KU496202	[24]
		Lygistopterus sanguineus	Greece	KU495979	KU496120	KU496120*	KU496182	[24]
	Calopterini	*Calopteron* sp.1	Panama	KT752118	KT751792	KT751792*	KT751951	[18]
		Calopteron sp.2	Ecuador	KT752129	KT751803	KT751803*	KT751962	[18]
	Conderini	*Conderis* sp.1	Laos	KT752099	KT751774	KT751774*	KT751933	[18]
		Conderis sp.2	Cambodia	KT752108	KT751783	KT751783*	KT751942	[18]
	Dihammatini	*Dihammatus* sp.1	Sumatra	KT752094	KT751770	KT751770*	KT751928	[18]
		Dihammatus sp.2	China	KT752097	KT751772	KT751772*	KT751931	[18]
	Dilophotini	*Dilophotes* sp.1	Borneo	DQ180992	DQ181214	--	DQ181368	[25]
		Dilophotes sp.2	Malaysia	KJ405048	KJ405242	KJ405242*	KJ405368	[26]

		Mesolycus qinlinganus	China	OP735349	OP729892	OP803897	OP803899	This study
		Mesolycus breviplatus	China	OP735348	OP729893	OP803896	OP803898	This study
	Erotini	*Eropterus* sp.	China	KT752046	KT751725	KT751725*	KT751883	[18]
		Lopheros sp.	Japan	KT752054	KT751733	KT751733*	KT751891	[18]
	Eurrhacini	*Eurrhacini* sp.1	Nicaragua	KT752119	KT751793	KT751793*	KT751952	[18]
		Eurrhacini sp.2	Ecuador	KT752120	KT751794	KT751794*	KT751953	[18]
	Lycini	*Lycostomus* sp.	China	DQ180981	DQ181203	--	DQ181357	[25]
		Lycus sp.	Zambia	KT751979	KT751662	KT751662*	KT751824	[18]
	Lyponiini	*Lyponia nigrohumeralis*	China	DQ180974	DQ181196	--	DQ181350	[25]
		Lyponia sp.	China	DQ181026	DQ181248	--	DQ181402	[25]
	Macrolycini	*Macrolycus* sp.1	Japan	EF143217	EF143232	--	EF143246	[27]
		Macrolycus sp.2	China	DQ180975	DQ181197	--	DQ181351	[25]
	Metriorrhynchini	*Broxylus kalamensis*	Sulawesi	KC538793	KC538414	KC538414*	KC538607	[5]
		Cautires sp.	Borneo	KC538632	KC538245	KC538245*	KC538437	[5]
	Platerodini	*Plateros* sp.1	Malaysia	KT751980	KT751663	KT751663*	KT751825	[18]
		Plateros sp.2	Taiwan	KT751999	KT751680	KT751680*	KT751841	[18]
	Slipinskiini	*Flagrax* sp.1	RSA	KT752059	KT751737	KT751737*	KT751894	[18]
		Flagrax sp.2	Cameroon	KT752060	KT751738	KT751738*	KT751895	[18]
	Thonalmini	*Thonalmus* sp.1	Cuba	KT752091	KT751768	KT751768*	KT751925	[18]
		Thonalmus sp.2	Dominican Republic	KT752093	KT751769	KT751769*	KT751927	[18]
Outgroup	Lyropaeini	*Lyropaeus* sp.	Malaysia	KT752072	KT751749	--	KT751906	[18]

Note:*indicates that *COI* and *COII* share the same accession number. **--** indicates no *COII* mitochondrial gene segments.

Table S4. The optimal partition schemes and the best-fit substitute models for the ML and BI analysis of gene segments dataset.

Partitions	Models	Genes
P1	GTR+I+G	nad3_pos1
P2	GTR+I+G	nad3_pos2
P3	HKY+G	nad5_pos3
P4	GTR+I+G	rrnL_pos1,rrnL_pos2,rrnL_pos3
P5	GTR+I+G	cox1_pos1,cox2_pos1
P6	GTR+I+G	cox1_pos2,cox2_pos2
P7	HKY+I+G	cox1_pos3,cox2_pos3
